# Gremlin‐1 Drives Tumour Vascularization by Promoting Endothelial Differentiation and Angiogenesis

**DOI:** 10.1111/jcmm.71188

**Published:** 2026-05-20

**Authors:** Stefania Mitola, Roberto Ronca, Marco Presta, Michela Corsini

**Affiliations:** ^1^ Department of Molecular and Translational Medicine University of Brescia Brescia Italy

## Abstract

Gremlin‐1 is a secreted antagonist of bone morphogenetic protein (BMP) signalling, highly expressed in various malignant tumours and is associated with poor prognosis. In addition to its established pro‐angiogenic activity, its potential role in endothelial differentiation in tumour contexts remains incompletely defined. Here, we investigated whether gremlin‐1 contributes to tumour vascularization by promoting both endothelial differentiation and vascular recruitment. The expression of gremlin‐1 in murine embryonic stem cells (ESCs) promoted tumoroid development enriched in mesodermal and endothelial lineages in vitro, as indicated by the upregulation of lineage‐specific markers and the presence of CD31‐positive vascular‐like networks. In vivo, gremlin‐1‐expressing ESCs generated larger teratomas with pronounced stromal expansion and increased vascularization, while retaining multilineage differentiation capacity. Using the chick chorioallantoic membrane (CAM) assay to discriminate donor‐derived vasculogenesis from host‐driven angiogenesis, we observed that gremlin‐1‐expressing grafts exhibited enhanced growth and vascularization. Species‐specific endothelial labelling revealed the presence of both ESC‐derived and host‐derived endothelial cells within vascular structure. Collectively, our findings identify gremlin‐1 as a regulator of tissue vascularization that integrates intrinsic endothelial differentiation with extrinsic angiogenic responses, a mechanism potentially relevant to vascular remodelling in several pathological conditions including tumour growth.

## 1 Introduction

Gremlin‐1 is a highly conserved, multifunctional soluble protein involved in numerous physiological and pathological processes, including organogenesis, tissue homeostasis, inflammation, fibrosis and tumour growth. By binding bone morphogenetic proteins (BMP2, BMP4 and BMP7) [1, 2], gremlin‐1 regulates cell signalling and thereby regulates key developmental and differentiation programmes [3]. In tumours, gremlin‐1 is frequently overexpressed within the tumour stroma, particularly in cancer‐associated fibroblasts, where it contributes to the establishment of a pro‐tumorigenic ‘niche’ [4]. By suppressing BMP‐driven differentiation, gremlin‐1 supports the maintenance of progenitor‐like states and promotes tumorigenesis in several malignancies including gastric, colorectal, glioblastoma and breast cancer [5, 6]. Gremlin‐1 also exerts BMP‐independent pro‐angiogenic activity by directly activating vascular endothelial growth factor receptor‐2 (VEGFR2) [7] and FGFR1 [8], and interacting with heparan‐sulphate proteoglycans [7, 9, 10, 11]. These activities have been primarily linked to endothelial cell activation and sprouting angiogenesis. However, whether gremlin‐1 also influences endothelial lineage commitment, thereby contributing to vascularization upstream of angiogenesis, remains largely unexplored. Here, we address this question by investigating the role of gremlin‐1 in endothelial differentiation within a tumour‐like context. Using a murine embryonic stem cell (ESC)–based teratoma model [12], we combined in vitro tumoroid formation assays with in vivo analyses of teratoma growth and vascularization. ESC‐derived teratomas, which recapitulate multilineage differentiation from all three germ layers [13, 14] provide a powerful system to dissect how developmental signalling pathways regulate lineage specification and tissue organization. We show that gremlin‐1 expression enhances teratoma growth and vascularization. Mechanistically, gremlin‐1 promotes endothelial differentiation of ESCs while simultaneously increasing the recruitment of host‐derived vasculature. These findings extend the current view of gremlin‐1 as a pro‐angiogenic factor by identifying a previously unappreciated role in modulating endothelial lineage commitment. Thus, gremlin‐1 emerges as a dual regulator of tumour vascularization, integrating vasculogenesis‐like processes with classical angiogenic responses within the tumour microenvironment.

## 2 Materials and Methods

### 2.1 Murine ESC Culture

Murine 1122 *fgfr1* ^+/−^ ESC (ESC^wt^) (Deng CX Gene dev 1994) adapted to grow without feeder cells were maintained in growth medium DMEM (Life Technologies, Grand Island, NY, USA) supplemented with 20% foetal bovine serum (Lonza Bioscience Basel, Switzerland), 0.1 mmol/L non‐essential amino acids (Life Technologies), 1 mmol/L sodium pyruvate (Life Technologies), 0.1 mmol/L β‐mercaptoethanol (Sigma‐Aldrich St. Louis, MO, USA) and 2 mmol/L L‐glutamine (Life Technologies) supplemented with 1000 U/mL mouse leukaemia inhibitory factor (LIF Chemicon, Millipore, Billerica, MA, USA). Murine 1122 *fgfr1* ^+/−^ ESC were transduced with lentiviral particles encoded for Lv‐r‐gremlin to stabilize the ESC^gremlin^ line.

### 2.2 In Vitro Tumoroids

Exponentially growing ESC cells were resuspended in LIF‐deprived medium and cultured in 30 μL of hanging drops (400 cells) for 2 days to allow cell aggregation. Then, aggregates were transferred onto 0.5% agarose‐coated dishes and grown for 5 days in complete medium w/o LIF. On Day 7, tumoroids were transferred into 8‐well ChamberTek (Nunc, Roskilde, Denmark) tissue culture plates to allow adhesion. Aggregates were fixed in PFA 4% in PBS and stained or collected for RNA extraction.

### 2.3 Mouse Teratoma Formation

7 × 10^6^ ESC^wt^ or ESC^gremlin^ cells were subcutaneously injected (s.c.) into the flanks of female NOD/SCID mice (*n* = 6 per group). Tumour volumes were monitored over time using callipers, and the volumes were calculated according to the formula V = (*D* × *d* ^2^)/2, where *D* and *d* are the major and minor perpendicular tumour diameters, respectively. At the experimental endpoint, tumours were harvested, weighed and processed for subsequent analyses. All in vivo experiments were approved by the local animal ethics committee (OPBA, Organismo Preposto al Benessere degli Animali, Università degli Studi di Brescia, Brescia, Italy) and conducted in accordance with national guidelines from the Italian ‘Ministero della Salute’.

### 2.4 Teratoma Growth in Eggs

White Leghorn eggs were incubated at 37°C in a humidified incubator. Grafting procedures were conducted as previously described [15, 16]. Briefly, on embryonic Day 4, a small window was opened in the eggshell. On Day 7, a plastic coverslip with a central opening was placed on the surface of the CAM. 2 × 10^6^ of LIF‐deprived ESCs were grafted onto the CAM in proximity to a well‐vascularized area. Ten days after implant, samples were collected (*n* = 7–17), excised and processed for histological or gene expression analyses.

### 2.5 Flow Cytometry

Tumoroids were dissociated, resuspended in FACS buffer (PBS supplemented with 2% FBS) and stained with APC‐conjugated anti‐CD31 and PacificBlue‐conjugated anti‐VEGFR2 antibodies (BioLegend, San Diego, CA, USA). Samples were acquired using the MACSQuant Analyzer (Miltenyi Biotec, Bergisch Gladbach, Germany) and analysed using FlowJo. Gating strategy included the exclusion of debris and doublets, followed by the analysis of CD31^+^/VEGFR2^+^ double‐positive cells. Data are representative of three independent experiments.

### 2.6 Histological Staining and Immunohistochemistry

Teratoma samples were fixed in PFA 4% or Zinc Fix, embedded in paraffin and sectioned. Sections were stained with haematoxylin and eosin (H&E) or subjected to antigen retrieval in citrate buffer (pH 6.0) at 95°C for 20 min, followed by incubation with the primary antibodies anti‐CD31 (Dianova, Hamburg, Germany), anti‐Nestin (Santa Cruz Biotechnology, Dallas, TX, USA), anti‐vWF (Dako, Glostrup, Denmark) and anti‐PECAM (Santa Cruz Biotechnology). Nuclei were counterstained with API (Sigma‐Aldrich). Images were acquired using an Axio Observer microscope equipped with Apotome.2 and with Plan‐Apochromat 63X/1,4 Oil DIC objective (Carl Zeiss), and analysed using the Zen software. CD31 and vWFpositive areas were quantified using the ImageJ software on at least 4 independent sections per samples (*n* = 4 biological replicates). For each section, multiple fields were analysed, and positive staining was expressed as a percentage of the total area. Thresholding parameters were kept constant across all conditions.

### 2.7 Western Blot Analysis

Conditioned media or total protein extract were separated by SDS‐PAGE and probed with antigremlin (R&D System, Minneapolis, MN, USA) followed by HRP‐conjugated secondary antibody (Santa Cruz Biotechnology). Signals were acquired using the ChemiDocTM Imaging System (Bio‐Rad, Hercules, CA, USA).

### 2.8 Gene Expression

Total RNA was extracted by Trizol reagent according to the manufacturer's instruction. 2 μg of RNA were retrotranscribed using MML‐V (Life Technologies). Quantitative real‐time PCR (qRT‐PCR) was performed using the ViiA7 Real‐Time PCR System (Life Technologies). Gene expression levels were normalized to reference genes (chicken or mouse GAPDH) and calculated using the ΔΔCt method. Data are presented as relative fold change compared with control conditions. Each experiment was performed in at least three independent biological replicates.

### 2.9 Statistical Analyses

Statistical analyses were performed using the statistical package Prism11 (GraphPad Software). Data are presented as mean ± SEM unless otherwise specified. Student's *t*‐test for unpaired data (2‐tailed) was used to test the probability of significant differences between two groups of samples. The significance level was set at *p < 0.05, **p < 0.01, ***p < 0.001 and ****p < 0.0001; ns = not significant.

## 3 Results

### 3.1 Gremlin Modulates the Expression of Stemness‐Associated Transcription Factors

**FIGURE 1:**
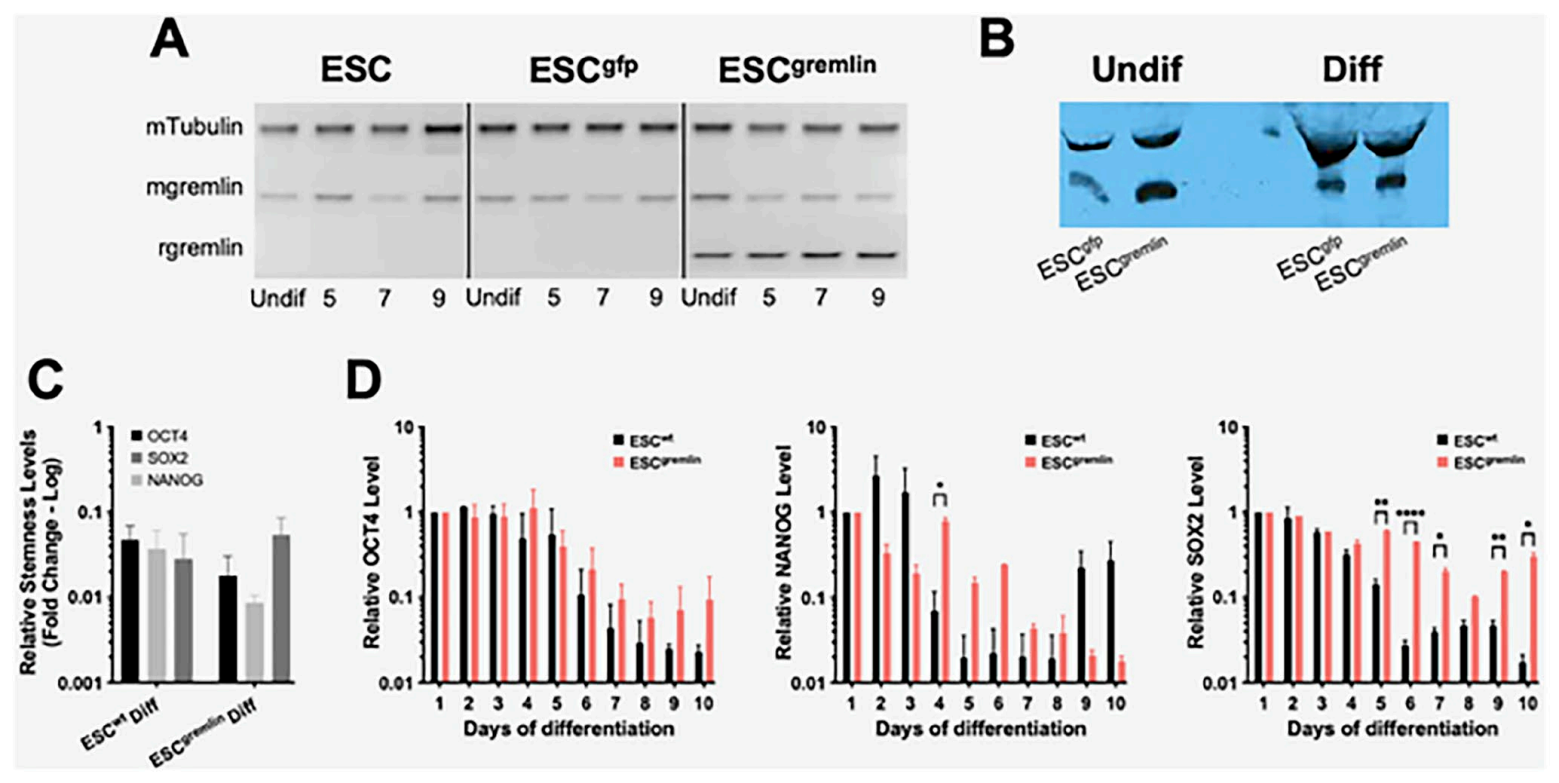
Gremlin modulates the expression of stemness markers in ESCs. (A) Semi‐quantitative PCR analysis of m‐gremlin and r‐gremlin both in the presence of LIF (Undif) and after 5, 7 and 9 days of differentiation. mTubulin has been used as a reference gene. (B) WB analysis of gremlin in the supernatant of Undif and Diff ESCs. (C) qPCR analysis of Oct4, Sox2 and Nanog expression upon 10 days of differentiation (Diff) of ESC^wt^ and ESC^gremlin^ cells. Data are expressed as relative‐fold‐change (log scale) ± SEM (*n* = 3). (D) Time‐course analysis of Oct4, Nanog and Sox2 expression during differentiation (Days 1–10) in ESC^wt^ (black bars) and ESC^gremlin^ (salmon bars). Data are expressed as relative‐fold‐change (log scale). Statistical significance: *p < 0.05, **p < 0.01, ****p < 0.0001.

Several evidences identified BMP2 and BMP4 as key inhibitors of cancer stem cell self‐renewal and drivers of their differentiation, for example, in glioblastoma stem cells [17]. Here, we investigated how gremlin‐1 expression, a BMP‐antagonist, influences ESC differentiation. To this end, ESCs were transduced with lentiviral particles encoding r‐gremlin (ESC^gremlin^) or EGFP (ESC^wt^). Gremlin expression was confirmed by PCR (Figure 1A) and Western blot (WB) analysis (Figure 1B). In the presence of LIF, gremlin‐1 overexpression did not affect ESC proliferation or their undifferentiated state.

As expected, LIF removal induced the formation of tumoroids with a progressive loss of stemness in both ESC^wt^ and ESC^gremlin^ cells within 10 days. qPCR analysis confirmed that all three stemness‐associated transcription factors, Oct4, Sox2 and Nanog, declined over time in both cell lines (Figure 1C). However, kinetic analysis revealed that gremlin expression markedly altered the timing of pluripotency exit. Indeed, ESC^gremlin^ cells retained higher Oct4 and Sox2 levels throughout differentiation while Nanog expression decreased in both lines, albeit with slightly altered dynamics (Figure 1D).

### 3.2 Gremlin Enhances Mesodermal Commitment and Endothelial Differentiation

**FIGURE 2:**
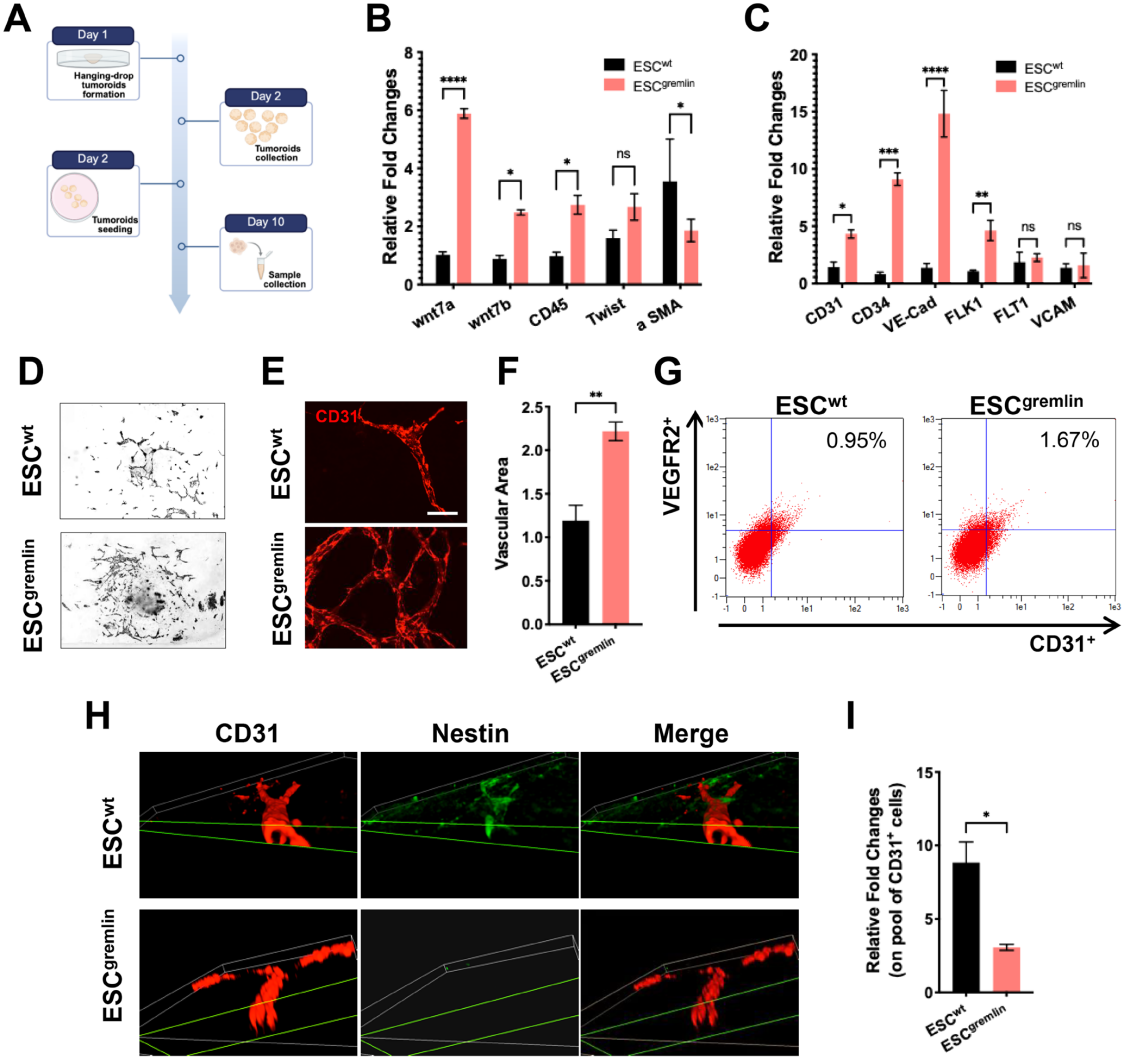
Gremlin promotes mesodermal commitment and vascular differentiation. (A) Schematic representation of in vitro tumoroid formation protocol. (B) qPCR analysis of mesodermal markers in ESC^wt^ and ESC^gremlin^ cells. Bars represent mean ± SEM (*n* = 4); *p < 0.05, ****p < 0.0001, ns = not significant. (C) qPCR analysis of endothelial markers in ESC^wt^ and ESC^gremlin^ cells. Bars represent mean ± SEM (*n* = 4); *p < 0.05; **p < 0.01; ***p < 0.001; ****p < 0.0001; ns = not significant. (D) Representative images of adhered tumoroids. (E) Representative confocal images of CD31 staining in ESC^wt^‐ and ESC^gremlin^‐derived tumoroids. Scale bars: 50 μm. (F) Quantification of CD31‐positive area. Bars represent mean ± SEM (*n* = 4); **p < 0.01. (G) Cytofluorimetric analysis of ESC^wt^ and ESC^gremlin^ cells. (H) Representative confocal z‐stack 3D reconstruction of CD31(red) and Nestin (green) co‐staining in ESC^wt^‐ and ESC^gremlin^‐derived tumoroids. (I) qPCR analysis of nestin expression in CD31‐positive cells. Bars represent mean ± SEM (*n* = 6); *p < 0.05.

Here, given the altered kinetics of pluripotency loss observed in ESC^gremlin^, we investigated whether gremlin‐1 expression affects lineage specification during in vitro differentiation using a tumoroid model. Tumoroids, generated by hanging drops of 400 cells, were differentiated for 10 days (Figure 2A). qRT‐PCR analysis revealed that gremlin enhances early mesodermal commitment as demonstrated by a marked induction of Wnt7a, Wnt7b, CD45 and *α*SMA, in ESC^gremlin^‐derived tumoroids (Figure 2B). Since endothelial lineages derived from mesoderm and given the mesoderm‐biased programme observed in ESC^gremlin^ cells, we assessed the effects of gremlin expression on endothelial specification. As expected, ESC^gremlin^‐derived tumoroids exhibited a robust upregulation of endothelial markers including CD31, CD34, VE‐Cad and FLK1, whereas FLT1 and VCAM levels remained comparable to ESC^wt^ (Figure 2C). These data suggest that gremlin did not globally activate endothelial programmes, but selectively reinforced specific transcriptional modules associated with endothelial differentiation. Consistent with these data, morphological and immunofluorescence analysis of adherent tumoroids highlighted that ESC^gremlin^ tumoroids developed broader stromal‐like CD31‐enriched areas. ESC^gremlin^‐derived tumoroids formed larger, denser and more interconnected vascular‐like networks than the sparse structures generated by ESC^wt^ (Figure 2D–F). This phenotype suggests that gremlin primes mesoderm formation and promotes the differentiation and expansion of endothelial‐committed progenitors. In line with these observations, flow cytometry demonstrated a higher proportion of CD31^+^/VEGFR2^+^ double‐positive cells in ESC^gremlin^‐derived tumoroids, indicating a substantial enhancement of endothelial commitment (Figure 2G).

3D confocal reconstruction revealed qualitative differences in endothelial maturation: CD31^+^ cells in ESC^wt^ cultures retained high nestin expression, a hallmark of immature endothelial progenitors, whereas CD31^+^ cells in ESC^gremlin^‐derived tumoroids showed markedly reduced nestin levels, consistent with a more advanced differentiation state (Figure 2H). This observation was supported by qRT‐PCR analysis of nestin expression normalized to CD31 levels, which confirmed reduced nestin expression in ESC^gremlin^‐derived endothelial cells (Figure 2I).

### 3.3 Gremlin Increases Teratoma Vascularization

**FIGURE 3:**
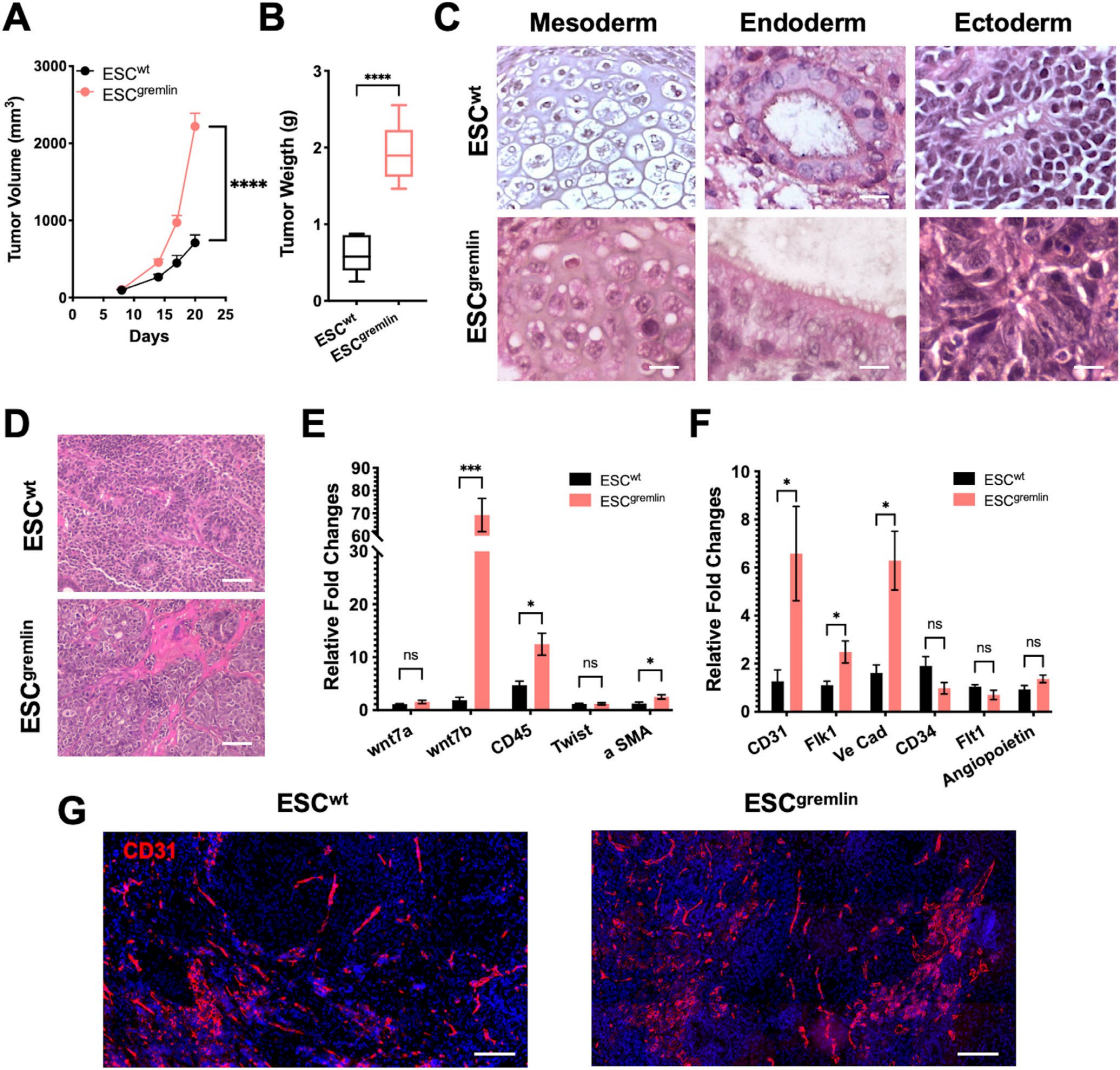
Gremlin promotes mesodermal and endothelial differentiation in ESC‐derived teratoma. (A) Growth curves of teratomas of ESC^wt^ and ESC^gremlin^ cells injected subcutaneously in immunocompromised mice (*n* = 6). Data are presented as mean ± SEM; ****p < 0.0001. (B) Tumour weight at endpoint of ESC‐derived teratomas. Data are presented as mean ± SEM (*n* = 6); ****p < 0.0001. (C) Representative H&E staining of FFPE teratoma sections showing the three germ layers (mesoderm, endoderm and ectoderm). Scale bars: 20 μm. (D) Representative H&E staining of FFPE teratoma sections. Scale bars: 100 μm. (E) qPCR analysis of mesodermal markers in ESC^wt^‐ and ESC^gremlin^‐derived teratomas. Data are shown as mean ± SEM; *p < 0.05, ***p < 0.001, ns = not significant. (F) qPCR analysis of endothelial markers. Data are shown as mean ± SEM; *p < 0.05, ns = not significant. (G) Representative immunofluorescence staining for CD31 (red). Nuclei are counterstained with DAPI (blue). Scale bars: 100 μm.

We then assessed the ability of ESC^wt^ or ESC^gremlin^ cells to form teratomas in immunocompromised female mice. ESC^gremlin^‐derived teratomas grew significantly faster and reached larger volumes and weight than ESC^wt^‐derived teratomas (Figure 3A,B), indicating that gremlin‐1 enhanced teratoma growth. Histological analysis revealed the presence of derivatives of all three germ layers (mesoderm, ectoderm and endoderm) in both groups, indicating that gremlin‐1 expression did not impair ESC pluripotency or their ability to generate multilineage teratomas (Figure 3C). Despite this, ESC^gremlin^‐derived teratomas were markedly enriched in mesoderm‐derived tissues, characterized by extensive stromal components and highly vascularized regions (Figure 3D) along with increased expression of mesoderm‐associated markers, including Wnt7b, CD45 and *α*SMA in ESC^gremlin^‐derived teratomas (Figure 3E). Consistently, endothelial markers such as CD31, Flk1 and VE‐cadherin were significantly upregulated in ESC^gremlin^ tumours (Figure 3F). CD31 staining further confirmed the presence of abundant and well‐organized vascular‐like structures in ESC^gremlin^‐derived teratomas, while ESC^wt^‐derived teratomas displayed sparse and discontinuous CD31^+^ areas, indicative of poorly structured vasculature. In addition, ESC^gremlin^‐derived teratomas contained clusters of CD31^+^ cells without clear vessel organization, suggesting the presence of endothelial progenitor or early stages of differentiation (Figure 3G).

Under these experimental conditions, it was not possible to discriminate between endothelial cells newly differentiated from ESCs and those recruited from the surrounding tissues via host‐driven angiogenesis.

### 3.4 Gremlin Simultaneously Induces Vasculogenesis and Angiogenesis

To determine whether the increased CD31^+^ area observed in ESC‐derived teratomas primarily reflected differentiation of ESC or also involved recruitment of host vasculature, we employed the chicken embryo chorioallantoic membrane (CAM) assay. This model enables a clear distinction between endothelial cells of murine origin, arising from ESC differentiation, and those derived from the avian host through angiogenic recruitment [15].

**FIGURE 4:**
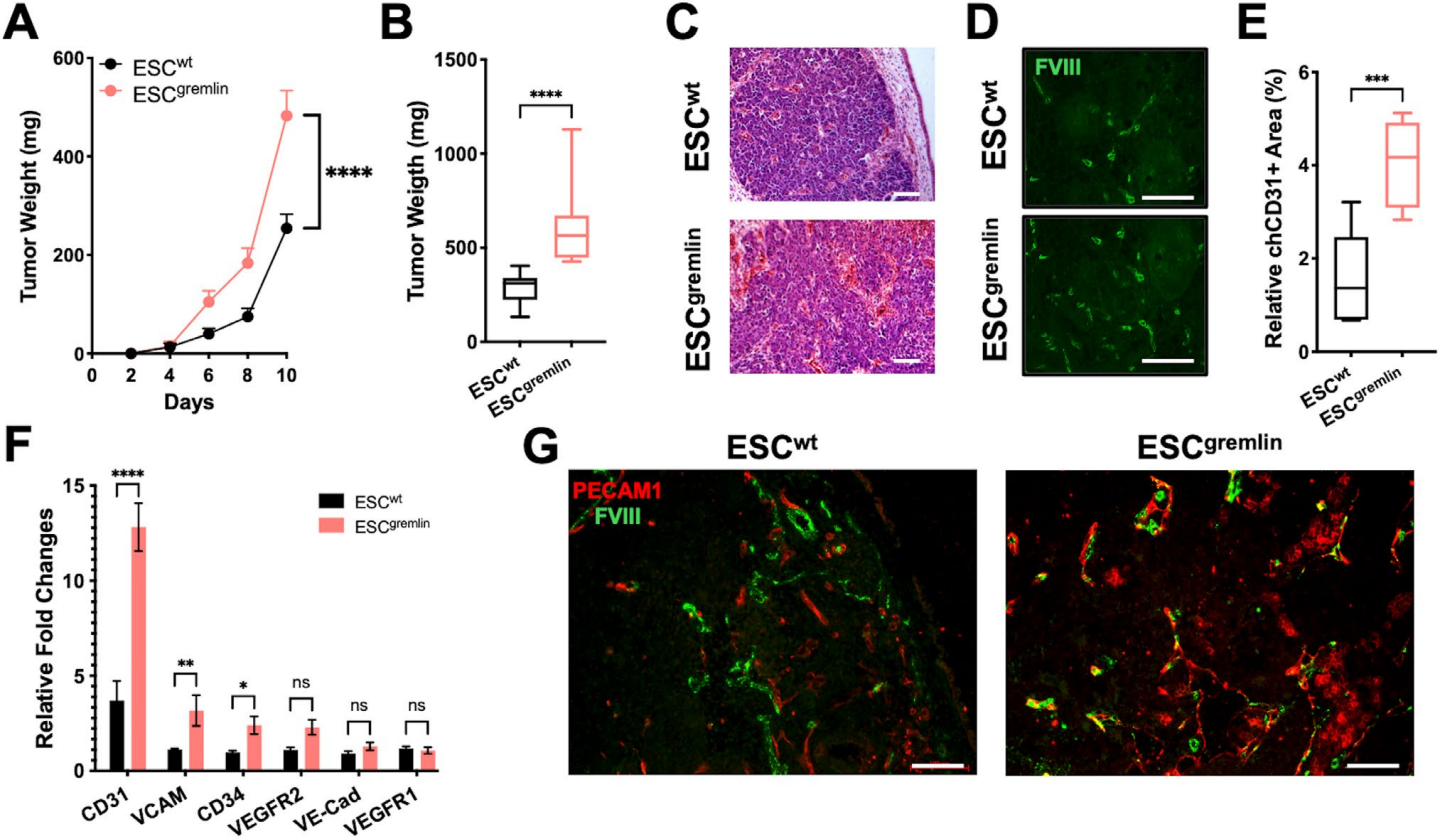
Gremlin enhances teratoma vascularization supporting both vasculogenesis and angiogenic processes (A) Growth curves of CAM teratoma derived from ESC^wt^ and ESC^gremlin^ (*n* = 17–7). Data are presented as mean ± SEM; ****p < 0.0001. (B) Tumour weight at endpoint of ESC‐derived teratomas. Data are represented as Min to Max box & whiskers plot (*n* = 7); ****p < 0.0001. (C) Representative H&E staining of tumour sections. Scale bars: 100 μm. (D) Representative immunofluorescence staining for FVIII (green). Scale bars: 100 μm. (E) Quantification of the FVIII positive area in ESC‐derived teratomas. Data are represented as Min to Max box and whiskers plot (*n* = 4); ***p < 0.001. (F) qPCR analysis of murine endothelial markers in ESC‐derived teratomas. Data are presented as mean ± SEM (*n* = 6); ****p < 0.0001; **p < 0.01; *p < 0.05; ns = not significant. (G) Representative immunofluorescence staining for FVIII (green) and PECAM1 (red) in ESC‐derived teratomas. Scale bars: 100 μm.

ESC^gremlin^‐derived tumours displayed significantly accelerated growth kinetics and reached larger sizes than ESC^wt^ counterparts (Figure 4A,B). Histological analysis (Figure 4C) and immunofluorescence staining (Figure 4D,E) revealed a higher degree of vascularization in ESC^gremlin^‐derived teratomas. To specifically assess the contribution of ESC‐derived endothelial differentiation, we analysed the expression of murine mesodermal and endothelial markers by qPCR supporting the concomitant differentiation of ESCs towards an endothelial‐like fate. ESC^gremlin^‐derived teratomas maintained higher levels of these markers than controls (Figure 4F), supporting enhanced differentiation of ESCs towards an endothelial lineage in vivo. In parallel, host‐derived angiogenic recruitment was evaluated using species‐specific endothelial markers. Co‐immunostaining for avian FVIII and murine PECAM1 revealed an increased presence of host‐derived endothelial components in ESC^gremlin^‐derived teratomas, along with areas of close association between murine and avian endothelial cells (Figure 4G).

Together, these data indicate that, in the CAM model, gremlin‐1 enhances both ESC‐derived endothelial differentiation and host‐derived angiogenic recruitment, allowing these two processes to be distinguished and independently assessed within the same experimental setting.

## 4 Discussion

Gremlin‐1 is a developmental regulator aberrantly reactivated in cancer and is associated with tumour progression and poor clinical outcome [18]. While its pro‐angiogenic activity has been primarily attributed to the activation of endothelial receptors and the recruitment of host vasculature [7, 19], our data indicate that gremlin‐1 also influences endothelial differentiation within a tumour‐like context.

In ESC‐derived tumoroids, gremlin‐1 was associated with increased CD31^+^/VEGFR2^+^ vascular structures and reduced nestin expression, consistent with enhanced endothelial specification and progression towards vascular maturation. Overall, gremlin‐1 emerges as a key regulator of mesoderm‐to‐endothelium differentiation, biasing pluripotent cells towards an endothelial lineage and contributing to the generation of tumour‐associated endothelial‐like cells from multipotent progenitors, thereby supporting tumour vascularization and growth.

The CAM assay provided a versatile, cost‐effective and 3R‐compliant complementary system to distinguish between intrinsic endothelial differentiation and host‐driven angiogenesis [15]. In this model, gremlin‐1‐expressing grafts displayed increased growth and vascularization, accompanied by upregulation of host‐derived endothelial markers, indicating enhanced angiogenic recruitment. Concurrently, the persistence of donor‐derived endothelial markers supports a contribution of ESC‐derived cells to the vascular compartment. The presence of vessels containing both avian and murine endothelial components suggests a close spatial association between these processes. Although the functional integration was not directly assessed in this study, previous observations in the same experimental settings have shown the presence of nucleated avian erythrocytes within chimeric vessels, indicative of vessel perfusion [15]. This supports the possibility that similar structures may be functionally connected to the host circulation.

Taken together, our observations support a model in which gremlin‐1 contributes to tumour vascularization by simultaneously promoting intrinsic endothelial differentiation and extrinsic angiogenic recruitment. Mechanistically, the pleiotropic activity of gremlin‐1 may reflect its ability to engage multiple signalling pathways. In addition to its established role as a BMP antagonist, gremlin‐1 can function as a ligand for receptors such as VEGFR2 and FGFR1. Inhibition of BMP signalling may sustain the maintenance of progenitor states and influence lineage commitment, while activation of VEGFR2 or FGFR1 may promote endothelial differentiation, survival and vascular remodelling. However, the relative contribution of these pathways was not specifically investigated in this study and remains to be determined [1, 20, 21].

Despite these limitations, our findings extend the current understanding of gremlin‐1 function by demonstrating that its pro‐vascular activity is not restricted to the activation of pre‐existing endothelial cells but also involves the modulation of cell fate decisions within the tumour microenvironment. This dual contribution may explain the association between gremlin‐1 expression, vascular remodelling and tumour progression observed [22, 23]. Given its combined effects on tumour cell proliferation, endothelial differentiation and angiogenesis, gremlin‐1 represents a potential therapeutic target. Further studies will be required to dissect the underlying signalling mechanisms and to determine whether targeting gremlin‐1 or its downstream pathways may provide therapeutic benefit in combination with existing antiangiogenic strategies. Targeting gremlin‐1 or its downstream signalling pathways, alone or in combination with antiangiogenic therapies, may provide a strategy to simultaneously impair tumour growth and disrupt vascular network formation, potentially overcoming resistance mechanisms [24, 25].

## Author Contributions

**Roberto Ronca:** conceptualization, investigation, writing – review and editing. **Marco Presta:** conceptualization, writing – review and editing. **Stefania Mitola:** conceptualization, investigation, funding acquisition, writing – original draft, writing – review and editing, project administration. **Michela Corsini:** conceptualization, investigation, writing – original draft, writing – review and editing, supervision.

## Funding

This work was funded by Associazione Italiana Ricerca sul Cancro AIRC (AIRC grant no. IG17276 to S.M.); AIRC fellowship for Italy (grant no. 26917 to M.C.); PNRR—CN3 ‘Sviluppo di Terapia Genica e Farmaci con Tecnologia ad RNA’ PNRR M4C2‐Investimento 1.4‐CN00000041 ‘finanziato dall'Unione Europea–NextGenerationEU’ (to S.M., M.P., R.R. and M.C.); Research funds from University of Brescia (ex 60%) 2024 to S.M., R.R. and M.C., and ‘5 per mille’ to M.C. This work was supported by grants from MIUR to Consorzio Interuniversitario Biotecnologie (CIB). Funding bodies did not have any role in designing the study, collecting, analysing and interpreting data or in writing the manuscript.

## Conflicts of Interest

The authors declare no conflicts of interest.

## Data Availability

All materials and relevant raw data described in this study are available upon request for non‐commercial research purposes.

## References

[1] L. Z. Topol , B. Bardot , Q. Zhang , et al., “Biosynthesis, Post‐Translation Modification, and Functional Characterization of Drm/Gremlin,” Journal of Biological Chemistry 275 (2000): 8785–8793. 10722723 10.1074/jbc.275.12.8785

[2] R. H. Church , A. Krishnakumar , A. Urbanek , et al., “Gremlin1 Preferentially Binds to Bone Morphogenetic Protein‐2 (BMP‐2) and BMP‐4 Over BMP‐7,” Biochemical Journal 466 (2015): 55–68. 25378054 10.1042/BJ20140771

[3] M. Ichinose , N. Suzuki , T. Wang , et al., “The BMP Antagonist Gremlin 1 Contributes to the Development of Cortical Excitatory Neurons, Motor Balance and Fear Responses,” Development 148 (2021): 148. 10.1242/dev.195883 PMC8313862 34184027

[4] J. Ren , M. Smid , J. Iaria , et al., “Cancer‐Associated Fibroblast‐Derived Gremlin 1 Promotes Breast Cancer Progression,” Breast Cancer Research 21 (2019): 109. 31533776 10.1186/s13058-019-1194-0 PMC6751614

[5] Y. Gao , S. De , and D. P. Brazil , “The Role of GREMLIN1, a Bone Morphogenetic Protein Antagonist, in Cancer Stem Cell Regulation,” Cells 14 (2025): 14. 10.3390/cells14080578 PMC12025430 40277903

[6] N. M. Elemam , A. I. Malek , E. E. Mahmoud , W. El‐Huneidi , and I. M. Talaat , “Insights Into the Role of Gremlin‐1, a Bone Morphogenic Protein Antagonist, in Cancer Initiation and Progression,” Biomedicine 10 (2022): 10. 10.3390/biomedicines10020301 PMC8869528 35203511

[7] H. Stabile , S. Mitola , E. Moroni , et al., “Bone Morphogenic Protein Antagonist Drm/Gremlin Is a Novel Proangiogenic Factor,” Blood 109 (2007): 1834–1840. 17077323 10.1182/blood-2006-06-032276

[8] C. Cheng , J. Wang , P. Xu , et al., “Gremlin1 Is a Therapeutically Targetable FGFR1 Ligand That Regulates Lineage Plasticity and Castration Resistance in Prostate Cancer,” Nature Cancer 3 (2022): 565–580. 35624341 10.1038/s43018-022-00380-3

[9] S. Mitola , C. Ravelli , E. Moroni , et al., “Gremlin Is a Novel Agonist of the Major Proangiogenic Receptor VEGFR2,” Blood 116 (2010): 3677–3680. 20660291 10.1182/blood-2010-06-291930

[10] E. Grillo , C. Ravelli , M. Corsini , et al., “Monomeric Gremlin Is a Novel Vascular Endothelial Growth Factor Receptor‐2 Antagonist,” Oncotarget 7 (2016): 35353–35368. 27174917 10.18632/oncotarget.9286 PMC5085234

[11] P. Chiodelli , S. Mitola , C. Ravelli , P. Oreste , M. Rusnati , and M. Presta , “Heparan Sulfate Proteoglycans Mediate the Angiogenic Activity of the Vascular Endothelial Growth Factor Receptor‐2 Agonist Gremlin,” Arteriosclerosis, Thrombosis, and Vascular Biology 31 (2011): e116–e127. 21921258 10.1161/ATVBAHA.111.235184

[12] M. Tatullo , B. Marrelli , C. Benincasa , et al., “Organoids in Translational Oncology,” Journal of Clinical Medicine 9 (2020): 9. 10.3390/jcm9092774 PMC7564148 32867142

[13] B. Wu , Y. Li , B. Li , et al., “DNMTs Play an Important Role in Maintaining the Pluripotency of Leukemia Inhibitory Factor‐Dependent Embryonic Stem Cells,” Stem Cell Reports 16 (2021): 582–596. 33636115 10.1016/j.stemcr.2021.01.017 PMC7940253

[14] J. T. Taiani , R. J. Krawetz , N. I. Zur Nieden , et al., “Reduced Differentiation Efficiency of Murine Embryonic Stem Cells in Stirred Suspension Bioreactors,” Stem Cells and Development 19 (2010): 989–998. 19775198 10.1089/scd.2009.0297 PMC3128313

[15] M. Corsini , C. Ravelli , E. Grillo , P. Dell'Era , M. Presta , and S. Mitola , “Simultaneously Characterization of Tumoral Angiogenesis and Vasculogenesis in Stem Cell‐Derived Teratomas,” Experimental Cell Research 400 (2021): 112490. 33484747 10.1016/j.yexcr.2021.112490

[16] T. Chastel , S. Filiberti , S. Mitola , R. Ronca , A. Turtoi , and M. Corsini , “Protocol for Performing Angiogenic and Tumorigenic Assays Using the in Ovo Chick Embryo Chorioallantoic Membrane Model,” STAR Protocols 6 (2025): 103663. 40022735 10.1016/j.xpro.2025.103663 PMC11919581

[17] K. Yan , Q. Wu , D. H. Yan , et al., “Glioma Cancer Stem Cells Secrete Gremlin1 to Promote Their Maintenance Within the Tumor Hierarchy,” Genes & Development 28 (2014): 1085–1100. 24788093 10.1101/gad.235515.113 PMC4035537

[18] H. Li , Y. Zhou , J. Xiao , and F. Liu , “A Comprehensive Prognostic and Immunological Implications of Gremlin 1 in Lung Adenocarcinoma,” Frontiers in Immunology 16 (2025): 1529195. 40066442 10.3389/fimmu.2025.1529195 PMC11891240

[19] C. Ravelli , S. Mitola , M. Corsini , and M. Presta , “Involvement of αvβ3 Integrin in Gremlin‐Induced Angiogenesis,” Angiogenesis 16 (2013): 235–243. 23053782 10.1007/s10456-012-9309-6

[20] M. Kišonaitė , X. Wang , and M. Hyvönen , “Structure of Gremlin‐1 and Analysis of Its Interaction With BMP‐2,” Biochemical Journal 473 (2016): 1593–1604. 27036124 10.1042/BCJ20160254 PMC4888461

[21] A. J. Tatsinkam , N. Rune , J. Smith , J. T. Norman , B. Mulloy , and C. C. Rider , “The Binding of the Bone Morphogenetic Protein Antagonist Gremlin to Kidney Heparan Sulfate: Such Binding Is Not Essential for BMP Antagonism,” International Journal of Biochemistry & Cell Biology 83 (2017): 39–46. 27979781 10.1016/j.biocel.2016.12.006

[22] L. R. Dutton , O. P. Hoare , A. M. B. McCorry , et al., “Fibroblast‐Derived Gremlin1 Localises to Epithelial Cells at the Base of the Intestinal Crypt,” Oncotarget 10 (2019): 4630–4639. 31384391 10.18632/oncotarget.27050 PMC6659803

[23] H. Davis , S. Irshad , M. Bansal , et al., “Aberrant Epithelial GREM1 Expression Initiates Colonic Tumorigenesis From Cells Outside the Stem Cell Niche,” Nature Medicine 21 (2015): 62–70. 10.1038/nm.3750 PMC4594755 25419707

[24] C. Quintavalle , F. Ingenito , G. Roscigno , et al., “Ex.50.T Aptamer Impairs Tumor‐Stroma Cross‐Talk in Breast Cancer by Targeting Gremlin‐1,” Cell Death Discov 11 (2025): 94. 40069570 10.1038/s41420-025-02363-6 PMC11897156

[25] G. C. G. Davies , N. Dedi , P. S. Jones , et al., “Discovery of Ginisortamab, a Potent and Novel Anti‐Gremlin‐1 Antibody in Clinical Development for the Treatment of Cancer,” MAbs 15 (2023): 2289681. 38084840 10.1080/19420862.2023.2289681 PMC10793705

